# Self-check with plaque disclosing solution improves oral hygiene in schoolchildren living in a children’s home

**DOI:** 10.1186/s13690-018-0296-y

**Published:** 2018-09-10

**Authors:** Yukiko Nagashima, Hideo Shigeishi, Eri Fukada, Hideaki Amano, Masahiro Urade, Masaru Sugiyama

**Affiliations:** 1 Department of Public Oral Health, Program of Oral Health Sciences, Graduate School of Biomedical & Health Sciences, Hiroshima University, 1-2-3 Kasumi, Minami-ku, Hiroshima, 734-8553 Japan; 2 Hyogo Dental Hygienist College, Hyogo, Japan; 3 Department of Maxillofacial Functional Development, Program of Oral Health Sciences, Graduate School of Biomedical & Health Sciences, Hiroshima University, Hiroshima, Japan

**Keywords:** Oral health guidance, Schoolchildren, children’s home

## Abstract

**Background:**

The effectiveness of an oral hygiene program for children living in a children’s home has been reported. However, to the best of our knowledge, no studies have evaluated the possible effects of self-checking of oral health among children residing in a children’s home. The objective of this study was to examine if self-checking using plaque disclosing solution improves oral hygiene in schoolchildren living in a children’s home.

**Methods:**

We enrolled nine schoolchildren (six girls) without untreated decayed teeth living in a children’s home in Japan. This preliminary study was designed as a 5-month program comprising group and individual instructions and self-checking using plaque disclosing solution. Paired t-test and Wilcoxon signed-rank test were used for statistical analysis to evaluate the change of Plaque Control Record (PCR) and Patient Hygiene Performance (PHP).

**Results:**

The mean PCR significantly decreased to 38.7% after 3 months of self-checking using disclosing solution compared with that before self-checking (i.e., at 1 month) (60.7%) (*P* < 0.01). PHP score significantly decreased to 1.4 at 4 months compared with that at baseline (2.8) and at 1 month (2.7) (*P* = 0.012 and *P* = 0.018). Improvement of oral hygiene status was evaluated according to the ratio of PCR at 4 months to that at 1 month. The average improvement ratio was 0.4 ± 0.35 (range: 0.0–1.0). Significant correlation was not found between improvement rate and school grade (*r* = 0.63, *P* = 0.070).

**Conclusions:**

Our results suggest that self-checking with disclosing solution may be effective in improving oral hygiene among schoolchildren at a children’s home.

## Background

According to the results of a survey conducted by the Ministry of Health, Labour and Welfare (2013), approximately 30,000 children are living in children’s homes in Japan [1]. Importance is attached to these children acquiring appropriate knowledge of oral health and establishing good oral health practice because they receive minimal family support for oral health. The oral health condition of children living in a children’s home has been previously reported [2–4]. Many children living in a children’s home have dental caries and show high levels of bacterial counts in their saliva [2]. In addition, the dental caries status of children living in a children’s home mainly depends on their worst child care environment in which they have ever lived [3]. The oral hygiene index and oral health knowledge of children living in a children’s home has been improved by oral health guidance [4].

Educational programs on oral health may provide short-term improvement of oral hygiene [5], indicating that simple conventional oral health guidance results in inadequate behavior change in participants. Additionally, conventional oral health guidance without effective procedure and a good relationship with participants are known to be ineffective over time [5, 6]. To the best of our knowledge, no studies have evaluated the possible effects of self-checking of oral health among children residing in a children’s home. Therefore, we examined whether self-checking with plaque disclosing solution improves oral hygiene in schoolchildren living in a children’s home.

## Methods

### Participants and methods

**Fig. 1:**
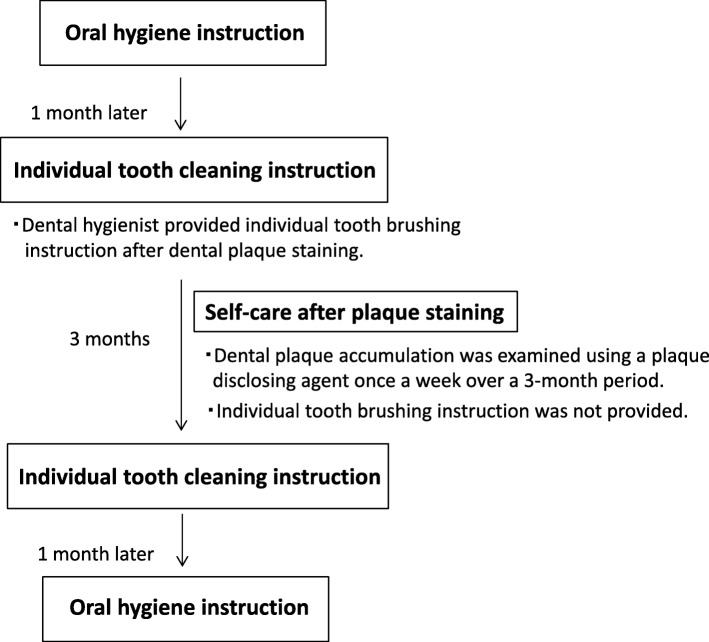
Flow diagram of the oral health instruction program in this study

We enrolled nine schoolchildren (six girls) without untreated decayed teeth living in a children’s home in Japan and provided them with oral health guidance from 2016 to 2017. The children were in 1st grade (*n* = 2), 3rd grade (*n* = 1), 4th grade (*n* = 3), 5th grade (n = 1) and 6th grade (n = 2). The study design was approved by the Ethical Committee of Hiroshima University (No. 1814) and informed consent was obtained from all participants and individuals in parental authority. The study was designed as a 5-month intervention program. The protocol for oral health guidance is described below and summarized in Fig. 1. All group and individual oral hygiene instructions were conducted by one dental hygienist.

Plaque Control Record (PCR) is a common and simple method of recording the presence of plaque on mesial distal, facial, and lingual surfaces at the gingival margin [7]. PCR index is calculated by dividing the number of plaque-containing surfaces by the total number of available surfaces. In addition, Patient Hygiene Performance (PHP) is useful for evaluating the extent of dental plaque on the tooth surface [8]. After applying disclosing solution to the teeth, six teeth surfaces are divided into five areas (i.e., three longitudinal thirds, distal, middle, and mesial; the middle third is subdivided horizontally into incisal, middle, and gingival thirds. Individual scores are obtained by totaling five subdivision scores per tooth surface and dividing by the number of tooth surfaces examined (score 0 to 5).

#### Oral hygiene instruction

Oral health education was performed (i.e., 20-min short play of oral hygiene to illustrate the etiology of dental caries and the optimal tooth cleaning method using large educational teeth model and toothbrush) by a dental hygienist. Oral hygiene instruction was given to the group after dental plaque staining. Data collection using a self-administered questionnaire was performed.

#### Individual tooth cleaning instruction

Individual tooth cleaning instruction was performed 1 month later. A dental hygienist provided individual tooth brushing instruction according to oral hygiene status after dental plaque staining. Data collection using a questionnaire was conducted.

#### Self-care after plaque staining

Dental plaque accumulation was examined using a plaque disclosing agent once a week over a 3-month period. PCR and PHP scores [7, 8] were recorded by a dental hygienist once a week after self-checking with disclosing solution following brushing. Individual tooth brushing instruction was not provided.

#### Individual tooth cleaning instruction after 3 months of self-care

A dental hygienist provided individual tooth brushing instruction after dental plaque staining. Data collection using a questionnaire was conducted.

#### Final oral hygiene instruction

Results of research were reported 1 month later.

### Statistical analysis

Paired t-test and Wilcoxon signed-rank test were used for statistical analysis, with *P* values less than 0.05 regarded to be statistically significant. Cochran’s Q test or Friedman test was used for statistical analysis of the results of the questionnaire about oral health. McNemar’s test was used to assesses the difference between paired proportions. Mann–Whitney U test was used as a nonparametric alternative to the independent t-test. Pearson’s correlation coefficient was also used for statistical analysis, which was performed using JMP® Pro12 software (SAS Institute Inc., Cary, NC, USA).

## Results

### Schoolchildren’s oral hygiene knowledge and practice before and after intervention

Responses to questions about oral health are summarized in Table 1 according to before and after intervention. Six of nine (66.7%) children brushed their teeth three times a day throughout this program. At baseline, 55.6% brushed using a mirror, rising to 66.7% at 1 month, then decreasing to 44.4% at 4 months. With regard to the toothbrushing item, 0, 0 and 66.7% used additional cleaning appliances besides a toothbrush at baseline, 1 month and 4 months, respectively, with an observed significant difference (*P* = 0.002, Cochran’s Q test). The proportion of schoolchildren used additional cleaning appliances significantly increased at 4 months compared with baseline (*P* = 0.031, McNemar’s test). Dental floss was an additional common cleaning appliance among six children at 4 months. The percentage of those replacing their toothbrush every month increased at 4 months compared with that at baseline, though the difference was not significant.

**Table 1:** Questionnaire about oral health

	Answers	At baseline	At 1 month	At 4 months	P value
Toothbrushing					
How often do you clean your teeth?	Never	0 (0%)	0 (0%)	0 (0%)	0.78
	Once a day	1 (11.1%)	0 (0%)	1 (11.1%)	
	Twice a day	2 (18.2%)	3 (33.3%)	2 (18.2%)	
	Three times a day	6 (66.7%)	6 (66.7%)	6 (66.7%)	
How long do you clean your teeth?	1 min	3 (33.3%)	3 (33.3%)	4 (44.4%)	0.23
	2 min	1 (11.1%)	0 (0%)	1 (11.1%)	
	3 min	2 (18.2%)	3 (33.3%)	2 (18.2%)	
	More than 5 min	1 (11.1%)	0 (0%)	0 (0%)	
	Not determined	2 (18.2%)	3 (33.3%)	1(11.1%)	
Do you clean your teeth in front of a mirror?	Yes	5 (55.6%)	6 (66.7%)	4 (44.4%)	0.65
	No	4 (44.4%)	3 (33.3%)	5 (55.6%)	
Toothbrushing item					
When do you usually change a toothbrush?	Every 1 month	2 (18.2%)	3 (33.3%)	5 (55.6%)	0.50
	Every 2–3 months	5 (55.6%)	3 (33.3%)	2 (18.2%)	
	Every 6 months	0 (0%)	0 (0%)	1 (11.1%)	
	Not determined	2 (18.2%)	2 (18.2%)	1 (11.1%)	
Do you use toothpaste?	Every time	8 (88.9%)	9 (100%)	9 (100%)	0.37
	Often	0 (0%)	0 (0%)	0 (0%)	
	Sometimes	1 (11.1%)	0 (0%)	0 (0%)	
	Never	0 (0%)	0 (0%)	0 (0%)	
Do you use fluoride toothpaste?	Yes	4 (44.4%)	6 (66.7%)	5 (55.6%)	0.47
	No	0 (0%)	0 (0%)	0 (0%)	
	I don’t know	5 (55.6%)	3 (33.3%)	4 (44.4%)	
Do you use additional tooth cleaning appliances besides a toothbrush?	Yes	0 (0%)	0 (0%)	6 (66.7%)	0.002
	No	9 (100%)	9 (100%)	3 (33.3%)	

Cochran’s Q test or Friedman test was used for statistical analysis

### Change of PCR and PHP score

**Fig. 2:**
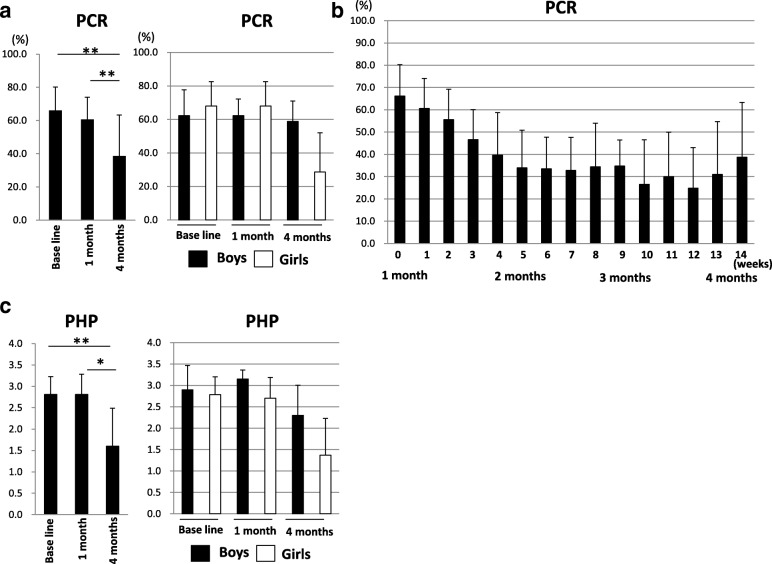
PCR and PHP score at baseline, at 1 month and at 4 months. *P* < 0.05 and *P* < 0.01 indicate statistically significant differences, labeled as * and ** respectively. **a** PCR score at baseline, at 1 month and at 4 months. Error bars represent the mean ± standard deviation. (**P < 0.01, paired t-test). **b** Change to PCR scores over a 3-month period of self-checking. **c** PHP score at baseline, at 1 month and at 4 months. Error bars represent the mean ± standard deviation. (*P < 0.05, Wilcoxon signed-rank test)

The mean PCR score slightly decreased to 60.7% at 1 month compared with those at baseline (66.1%). Then a significant decrease to 38.7% was observed after 3 months of self-checking using disclosing solution as compared with that at baseline and at 1 month (*P* = 0.009 and *P* = 0.007, respectively, paired t-test) (Fig. 2a). The mean PCR score was lower in girls than boys at 4 months, but no significant difference was observed. Interestingly, the mean PCR score gradually decreased throughout the first 5 weeks during the 3-month self-checking period, but no characteristic changes were later evident (Fig. 2b). PHP score slightly decreased to 2.7 at 1 month compared with at baseline (2.8), and then significantly decreased to 1.4 at 4 months compared with that at baseline and at 1 month (*P* = 0.012 and *P* = 0.018, respectively; Wilcoxon signed-rank test) (Fig. 2c). The mean PHP was lower in girls than in boys at 4 months, though a significant difference was not found.

### Correlation between improvement of oral hygiene and school grade

Improvement of oral hygiene status was evaluated according to the ratio of PCR at 4 months to that at 1 month (Table 2). The average of the improvement ratio was 0.40 ± 0.35 (range: 0.0–1.0). Significant correlation was not found between improvement rate and school grade (*r* = 0.63, *P* = 0.070). In addition, a significant association was not found between improvement ratio of PHP score and school grade (*r* = 0.39, *P* = 0.30).

**Table 2:** Correlation between school grade and improvement ratio of PCR and PHP

School grade	Improvement ratio of PCR	Improvement ratio of PHP
1	−0.028	0.067
1	0.068	0.64
3	0.48	0.48
4	0.32	0.46
4	0.11	0.0
4	0.57	0.26
5	1.00	1.00
6	0.22	0.57
6	0.83	0.67

## Discussion

It is vital to establish good oral health behaviors in childhood to ensure maintenance of good oral health in later life. Therefore, oral hygiene instruction is thought to be necessary for schoolchildren to improve oral health behaviors and prevent oral disease such as dental caries and gingivitis. Even though individual guidance can improve children’s oral hygiene [9], guidance without effective procedure may not be enough to promote their oral health status continuously [6]. In addition, long-term oral health programs may be effective in improving oral health-related attitude [10], suggesting that intervention duration is another significant factor for schoolchildren. Therefore, we examined whether self-checking using plaque disclosing solution over 3 months had a positive effect on oral hygiene status and behavior in schoolchildren. Because children living in the same children’s home tend to have similar dietary and lifestyle habits, relatively small individual differences in lifestyle habit concerning oral hygiene was expected. In addition, to the best of our knowledge, no studies have evaluated the possible effects of self-checking of oral hygiene among children in a children’s home. We thus investigated the effect of oral health guidance on those children.

Our results suggest that oral health guidance, including individual brushing instruction and self-checking over 3 months, significantly improves oral hygiene. Importantly, schoolchildren could visually notice plaque on tooth surfaces by themselves. Therefore, self-checking using plaque disclosing solution may have motivated schoolchildren to improve their dental practices. In addition, long-term programs (i.e., 5 months) may have been the primary reason for the observed good oral hygiene status of schoolchildren by maintaining their motivation and oral health practice. With regard to dental hygiene knowledge, the number of children using both a toothbrush and dental floss and frequency of toothbrush use was increased at 4 months. These results indicate that our program may have affected their oral health behavior and advanced knowledge of tooth brushing among schoolchildren.

Older schoolchildren showed a greater improved PCR score compared with younger schoolchildren at 4 months, indicating that self-checking is more effective in children of higher grade. In addition, girls showed better PCR and PHP score compared with boys. Thus, girls may have improved their oral hygiene more effectively than boys. These results were consistent with a previous report examining the correlation between age/gender and children’s oral hygiene status [11].

The number of schoolchildren in this study was small; therefore, further investigation using a larger number of participants is necessary to clarify the significance of our oral health guidance in schoolchildren. Furthermore, a control group without self-checking in oral health guidance should be included in a future study to demonstrate the significance of self-checking using plaque disclosing solution for oral health guidance.

## Conclusions

Despite the absence of a control group in this study, self-checking with plaque disclosing solution may play a significant role in improving the oral hygiene of schoolchildren at a children’s home. Further modified oral health guidance will be required to develop and maintain good oral health and behaviors in schoolchildren.

## Abbreviation

PCR: Plaque control record
PHP: patient hygiene performance
